# Effects of hyperthermia on DNA repair pathways: one treatment to inhibit them all

**DOI:** 10.1186/s13014-015-0462-0

**Published:** 2015-08-07

**Authors:** Arlene L. Oei, Lianne E. M. Vriend, Johannes Crezee, Nicolaas A. P. Franken, Przemek M. Krawczyk

**Affiliations:** Laboratory for Experimental Oncology and Radiobiology (LEXOR), Center for Experimental and Molecular Medicine, Academic Medical Center, University of Amsterdam, 1105 AZ Amsterdam, The Netherlands; Van Leeuwenhoek Centre for Advanced Microscopy (LCAM)-AMC, Department of Cell Biology and Histology, Academic Medical Center, University of Amsterdam, Meibergdreef 15, 1105 AZ Amsterdam, The Netherlands; Department of Radiotherapy, Academic Medical Center, University of Amsterdam, 1105 AZ Amsterdam, The Netherlands

**Keywords:** Hyperthermia, DNA damage, DNA repair, Chemotherapy

## Abstract

The currently available arsenal of anticancer modalities includes many DNA damaging agents that can kill malignant cells. However, efficient DNA repair mechanisms protect both healthy and cancer cells against the effects of treatment and contribute to the development of drug resistance. Therefore, anti-cancer treatments based on inflicting DNA damage can benefit from inhibition of DNA repair. Hyperthermia – treatment at elevated temperature – considerably affects DNA repair, among other cellular processes, and can thus sensitize (cancer) cells to DNA damaging agents. This effect has been known and clinically applied for many decades, but how heat inhibits DNA repair and which pathways are targeted has not been fully elucidated. In this review we attempt to summarize the known effects of hyperthermia on DNA repair pathways relevant in clinical treatment of cancer. Furthermore, we outline the relationships between the effects of heat on DNA repair and sensitization of cells to various DNA damaging agents.

## Introduction

Hyperthermia – treatment above temperatures that are physiologically optimal – affects cells and tissues on countless levels, by directly altering the physical properties of cellular components and by evoking counteractive cellular responses. Among other effects, heat causes DNA, protein and membrane damage, interferes with cell cycle, DNA and protein synthesis and may result in cell death, either directly or by triggering apoptotic pathways [1–5].

Early research demonstrated that except for the cytotoxic potential, hyperthermia can sensitize cells to DNA damaging agents. Indeed, elevated temperature, applied in combination with various anti-cancer drugs or radiation, has been shown to eradicate transformed cells in vitro and to inhibit tumor growth in animal models [6–13]. It was also speculated, based on results obtained using biochemical methods, that heat may induce DNA damage directly [14–16]. In the subsequent decades, an extensive body of data confirmed that hyperthermia is a powerful sensitizer to many agents that interfere with DNA metabolism or cause DNA damage, suggesting that it might directly interfere with DNA repair. However, how hyperthermia sensitizes cells to DNA damaging agents remained unclear. This changed gradually during the last two decades. With the introduction of advanced fluorescence imaging and molecular biology techniques in the 1990s came deeper understanding of DNA repair networks that, in turn, facilitated interpretation of results. During the last decade a number of important findings cemented the position of hyperthermia research within the DNA repair field and first large clinical trials clearly demonstrated the benefits of hyperthermia as adjuvant in clinical treatment of cancer [17–19] and stimulated research and development of new treatment approaches, such as hyperthermia-mediated drug release [20]. Nevertheless, the effects of hyperthermia on DNA repair are still not sufficiently understood.

It is clear that cytotoxic or sensitizing effects of hyperthermia cannot be attributed to deactivation of a single DNA repair mechanism, but rather to influencing many pathways, on multiple levels. Although this may hamper the interpretation of experimental data, the pleiotropic effects of heat on DNA repair may be extremely beneficial in the clinical settings. Therefore, understanding how heat interacts with the DNA repair networks will help in improving the existing and designing novel (combination) therapies. This review attempts to categorize the influence of hyperthermia on the known DNA repair pathways, with special attention to those pathways relevant in cancer treatment. Due to space and subject limitations, the effects of hyperthermia on other metabolic pathways or tissues and organs are not discussed, even though they might be as (or more) important in anti-cancer treatments.

One important factor that generally confounds analysis of available literature data is that different thermal doses are used in different studies. The thermal dose depends on the temperature and duration of treatment so that thermal dose equivalent at a given temperature can in principle be calculated using Arrhenius equations. For instance, cumulative equivalent minutes at 43 °C (CEM_43_) can be calculated to compare results of experiments or clinical treatments performed at different temperatures [21]. Accordingly, except for relatively high (>45 °C) temperatures, in principle the effects observed at a given temperature can be achieved by using a lower temperature and longer incubation time. We therefore intentionally do not limit our review to clinically relevant temperatures (<43 °C). Such approach allows inclusion of a broader spectrum of hyperthermia effects but caution should be exercised when directly comparing results of experiments performed at different temperatures.

### Direct induction of DNA damage by hyperthermia

It is generally accepted that hyperthermia inhibits DNA repair. However, the fundamental question whether hyperthermia directly induces DNA damage has not been definitively answered. Early studies showed that hyperthermia may induce DNA breaks and chromosomal aberrations, either by causing protein denaturation or by interfering with replication [14–16, 22–25]. Increased levels of 8-oxoguanine, apurinic sites and deaminated cytosines have also been detected after heat treatment [26]. Other studies showed that hyperthermia does not cause DNA damage in absence of additional stimuli. However, heat seemingly increased the levels of single strand breaks (SSBs) and double strand breaks (DSBs) during processing of damage induced by ionizing radiation, possibly by impairing the repair of corrupted bases [24, 27, 28]. Nearly a decade later it was reported that heat (>41.5 °C) triggers focal phosphorylation of histone H2AX, similar to the formation of the so-called ionizing radiation induced foci (IRIF) [29–31] that are generally considered to occur in response to DSBs [32, 33]. Moreover, this response was observed at relatively mild temperatures and the number of foci was proportional to thermal dose and cell killing. Interestingly, the induction of phospho-H2AX (γH2AX) foci was suppressed by prior heat treatment, resembling the known phenomenon of thermotolerance [34]. The authors suggested that the proposed induction of DSBs by hyperthermia may not be direct, but rather a result of nicks induced in close proximity on opposing DNA strands [29].

Later studies confirmed the induction of γH2AX and MDC1 foci by hyperthermia (43–45.5 °C) and showed its dependence on DSB signaling factor ATM [30, 35–38]. However, hyperthermia-induced foci did not recapitulate all characteristics of IRIF in that they failed to co-localize with 53BP1 or SMC1. Importantly, neither DNA damage, nor chromosome aberrations were detected in these studies, suggesting that heat may induce chromatin changes that in turn trigger DNA damage responses (DDR) in the absence of actual DNA damage [35]. Such triggering by different stimuli has indeed been observed earlier [39–42].

Adding to the debate, Velichko and colleagues recently reported that two different patterns of γH2AX foci can be discerned in hyperthermia-treated cells (42–45.5 °C): the larger IRIF-like foci in G_1_- and G_2_-phase cells and the smaller but more numerous foci in S-phase cells [43]. Even more surprisingly, hyperthermia-induced DSBs were detected in heated G_1_/G_2_ cells but not in S-phase cells, while the inverse was true for SSBs. Furthermore, the authors demonstrated inhibitory effects of heat on replication fork progression. The absence of DSBs in cells heated in S-phase can be caused by suppression of replication fork progression that might, in turn, prevent DSB formation [44]. The S-phase specific ‘protective’ foci may thus mark sites of stalled replication forks that are not yet converted to DSBs. On the other hand, the foci in non-S phase cells could mark DSBs that were directly induced by heat or, alternatively, persistent DSBs [45] that were unmasked by heat-related chromatin changes. This latter explanation may be difficult to prove since only a limited number of persistent DSBs have been observed earlier while hyperthermia can induce a large number of foci. Moreover, the chromatin domains containing persistent DSBs are decorated with 53BP1 [45], in contrast to heat-induced foci [35].

Clearly, the question whether heat directly induces DSBs is far from resolved. The majority of studies failed to detect DSBs or chromosome aberrations in heated cells by direct methods [46]. Most reports that did confirm induction of DSBs by heat rely on indirect assays such as phosphorylation of H2AX or accumulation of repair-related proteins. Some other studies confirmed DSB induction by direct methods and showed that phosphorylation of H2AX correlates with cell killing and thermotolerance. More sensitive and specific methods to directly detect DNA DSBs and SSBs may be required to settle the long-standing dispute.

### DNA damage signaling and cell cycle checkpoint activation

Even though it is far from certain whether hyperthermia can directly damage DNA, the triggered signaling resembles, to some extent, the responses caused by DNA damaging agents (see also previous section). In mammalian cells, such signaling can initiate checkpoints which interrupt the cycle progression to provide time for DNA repair and are thus essential for the maintenance of genomic integrity [47]. The mammalian checkpoints started in response to DNA damage are managed by the two master kinases, ATM and ATR [48, 49]. ATM is thought to be activated, with help of the the MRN (MRE11/RAD50/NBS1) complex, by DSBs, mainly in G_1_-phase [47, 50]. ATR chiefly responds to exposed single stranded DNA at stalled replication forks in S-phase, in a manner that is at least partly dependent on ATM [51, 52]. Both ATM and ATR, as well as the DNA-PK kinase, phosphorylate histone H2AX (see also previous section) and many other repair factors in chromatin domains surrounding the damaged DNA. This, in turn, triggers accumulation of multiple DNA repair-related proteins at damaged chromatin, further propagation of the damage signal and activation of the appropriate cell cycle checkpoints via mechanisms dependent on Chk1, Chk2, p53, CDC25a, WEE1 and other factors [47].

Mammalian cells display varying thermosensitivity, depending on the cell cycle phase in which they were heated [53, 54]. In general, G_1_-phase cells are relatively heat resistant and do not show any damage upon microscopic examination. S-phase cells are more sensitive and chromosomal damage is observed [55, 56]. The highest heat sensitivity can be observed during the M-phase, with damage of cellular mitotic apparatus leading to inefficient cell division and polyploidy. Hyperthermia induces a ‘slow mode of cell death’ in S- and M-phase, while cells heated during G_1_-phase may enter a ‘rapid mode of death’ [54, 57]. These variations in sensitivity between the different cell cycle phases suggest diversity of molecular mechanisms regulating cell death following hyperthermia, which may indicate involvement of various checkpoint mechanisms [53, 58, 59]. However, the influence of hyperthermia on cell cycle progression is not well understood. Early studies showed increase in length of all phases and arrest at the G_1_/S transition [60–62], but the underlying mechanisms were unclear. More recent work confirmed activation of cell cycle checkpoints by 42–46 °C heating [63] and implicated activation of ATM and a subset of its downstream targets, including p53, independently of the MRN complex [35, 64] (see also previous section). Another study reported activation of p53 via the thioredoxin-dependent redox state and modulation of checkpoint regulators Gadd45a and Cdc2 at 41 °C [65]. On the other hand, hyperthermia seems to disturb early steps in cellular responses to radiation-induced damage, as delayed formation of 53BP1 foci and phosphorylation of SMC and Chk2 have been reported after treatment at 43 °C [66]. This may be surprising, since ATM directly phosphorylates Chk2 in response to heat [64, 67]. Thus, while heat treatment alone may activate cell cycle checkpoints via the ATM kinase, it can apparently also delay signaling triggered by exogenously induced DNA damage.

ATR and Chk1 are also activated by heat (42.5-45 °C), reportedly to a larger extent than the ATM/Chk2 branch of the DDR, and the ensuing signaling cascade causes G_2_/M arrest which can be abrogated by inhibition of Chk1 [67, 68]. Chk1 activation is dependent on Rad9, Rad17, TopBP1 and Claspin, which play important roles in activation of ATR at stalled replication forks [69]. However, similarly to heat-induced ATM signaling, not all targets of ATR are activated as neither FancD2 monoubiquitination nor RPA32 phosphorylation were observed [67]. Hyperthermia (43–48 °C) also influences S-phase progression by directly inhibiting multiple processes related to replication [70–72]. Contributing to these effects is the release of nucleolin from the nucleolus which stimulates RPA-nucleolin interactions and may thus limit RPA involvement in replication. It seems feasible that this could, in turn, cause slowing or collapse of replication forks and initiate ATR signaling. In the context of S-phase, the activation of cell cycle checkpoint might therefore be a protective response mitigating the effects of hyperthermia on replication progression. Indeed, mammalian cells are exceptionally sensitive to heat in S-phase and at least part of hyperthermia-related cytotoxicity observed in S-phase cells can be alleviated by inhibition of replication [44]. Both ATM and ATR, as well as DNA-PK, seem to propagate damage signaling in response to heat by phosphorylating histone H2AX [43], with ATM/ATR responding to the presumed heat-induced DNA damage and DNA-PK reacting to heat-induced replication arrest (see also previous section). Intriguingly, the DNA-PK (but not ATR)-mediated H2AX phosphorylation may protect replication forks from collapse and DSB formation [43].

Based on the effects described above, it could be predicted that hyperthermia sensitizes cells to agents that interfere with cell cycle (checkpoints). Indeed, after treatment with hyperthermia (42 °C), antimitotic drugs like paclitaxel, nocodazole or Aurora A inhibitor showed increased toxicity. However, this was not due to activation of cell cycle checkpoints but, surprisingly, due to abrogation of the M checkpoint and forced mitotic exit, resulting in mitotic catastrophe [73]. Although hyperthermia (at 41.5 °C) also stimulates mitotic catastrophe in X-irradiated cells, this is accompanied by strengthening, rather than weakening, of radiation-induced S and G_2_ checkpoints [74]. It is not clear what mechanisms are responsible for the increased heat sensitivity of M-phase cells [75], but DNA damage repair is limited in this phase [76], which could explain heat-sensitivity if DNA damage is directly caused by hyperthermia.

Concluding, the effects of hyperthermia on cell cycle progression and checkpoint activation seem to be mediated, to a large extent, by ATM and ATR, the two factors that primarily regulate checkpoints in response to DNA damage. This could indicate that heat induces DNA damage, which in turn activates the DDR cascade. The preferential activation of ATR/Chk1 [67] suggests that, if DNA lesions are indeed induced by heat, they might be related to inhibited or corrupted replication forks. On the other hand, the differences in patterns of signaling initiated by heat, as compared to signaling triggered by direct DNA damage, may suggest involvement of other unidentified mechanisms, such as those related to chromatin changes [77].

### Excision repair

Excision repair in mammalian cells encompasses mechanisms that remove corrupted bases or nucleotides and fix DNA mismatches. Excision repair can be subdivided into base excision repair (BER), nucleotide excision repair (NER) and mismatch repair (MMR), with BER and NER proceeding via a SSB intermediate and thus sharing the final steps with SSB repair mechanisms.

BER constitutes the main pathway for the repair of DNA lesions induced by oxidizing or alkylating agents, as well as by endogenous metabolic activities. BER is active throughout the cell cycle and executed by a number of proteins that include DNA glycosylases, apurinic/apyrimidinic endonucleases, phosphatases, phosphodiesterases, kinases, polymerases and ligases [78]. BER is initiated by various glycosylases which recognize and remove the damaged bases and create abasic (AP) DNA sites. AP endonucleases (APE1 in human cells) then recognize and cleave AP sites and recruit DNA polymerases to restore the gaps, via a SSB intermediate, where BER and SSB repair pathways converge. Tens of thousands of damaged bases per day must be fixed in a mammalian cell, thus BER has evolved as a fast and efficient mechanism of paramount importance for maintaining the genomic integrity [78].

It has been suggested that BER might be the main target of heat at temperatures above 43.0 °C [79, 80]. Indeed, a measurable inhibition of base excision in X-irradiated cells was observed after hyperthermia [81]. Additionally, although hyperthermia treatment (43–45 °C) did not induce DNA damage by itself, it increased the amount of damaged bases and DSBs in X-irradiated cells [27], possibly by inhibiting BER and thus indirectly stimulating conversion of damaged bases to DSBs. Hyperthermia (>41.5 °C) has also been shown to affect the activity of DNA Pol β, an important BER factor [82–86]. However, the lack of correlation between Pol β activity and hyperthermic cell killing has also been reported [87]. In contrast to Pol β, effects of hyperthermia on its partner XRCC1 that is involved in later steps of BER and in SSB repair have not been explored, but it is intriguing that the molecular chaperone HSP90, part of the cellular responses to heat shock, influences DNA repair by regulating interactions between Pol β and XRCC1 [88]. It could be speculated that upon hyperthermia treatment HSP90 is required to chaperone its other client proteins, which could result in decreased mediation of XRCC1-Pol β interactions. Recently it has been confirmed that mild hyperthermia (42 °C) directly impairs BER, at least partially by affecting the cellular glycosylase activities [89]. In particular, hyperthermia inactivates 8-oxoguanine DNA glycosylase (OGG1) by depleting it from the nucleus and eliciting its proteasome-mediated degradation. The inhibition of OGG1 then likely contributes to heat-induced radio- or chemosensitization.

On the other hand, siRNA-mediated downregulation of AP endonuclease (APE1), a critical BER enzyme, failed to influence hyperthermic radiosensitization in HeLa cells, suggesting that BER is not affected by 41.5 °C incubation [90]. It should be noted, however, that only about 70 % downregulation of APE1 was achieved in this study and the residual protein levels might be sufficient to sustain (partial) BER activities. Moreover, the contribution of APE1 to cellular radiation responses is unclear and while some studies show that decreased APE1 levels correlate with increased radiosensitivity others show the opposite effects [90, 91].

Nucleotide excision repair (NER) is involved in excision mechanisms that remove DNA damage like pyrimidine dimers and (6–4)photoproducts [92]. The influence of hyperthermia on NER has not been extensively explored, but one study showed reduced NER-associated strand incision and considerably delayed repair of thymidine dimers in cultured human fibroblasts and keratinocytes heated at 43 °C. Additionally, the repair of UV-B-damaged plasmid DNA was lower if the transfected cells were exposed to heat [93].

One argument supporting the notion that hyperthermia interferes with NER stems from studies on sensitization to platinum-based compounds. Cisplatin and its derivatives, used widely in clinical cancer treatment, produce DNA interstrand cross-links that can be either repaired by the NER machinery or, after conversion to DSBs, by replication-coupled repair [94–96]. A wide body of evidence indicates that 39–43 °C hyperthermia sensitizes cells to cisplatin [97–100], suggesting that NER may indeed be among heat targets. One study compared hyperthermia-mediated (40–41 °C) sensitization to cisplatin in cells lacking the major NER factor XPA with wild-type cells [101]. Results showed comparable sensitization in both cell lines, leading to suggestion that NER plays no major role in this process. However, cisplatin-induced DNA lesions can also be repaired by pathways other than NER [95], which could explain these results, although these other pathways can also be affected by heat. Among modulatory effects of hyperthermia on cellular responses to cross-linkers is also suppression of cisplatin-induced XPC and XPA, as shown in human epithelial ovarian cancer xenografts incubated at 43 °C [102].

The effects of hyperthermia on MMR are even less explored. It has been shown that MMR factors hMLH1 and hMSH2 translocated from the nucleus into the cytoplasm in response to 41–42 °C heat shock [103]. This study also showed, by applying comet assay, that hyperthermia induces DNA damage. Surprisingly, in heat-shocked MMR-deficient cells less DNA damage was detected than in wild-type counterparts, for up to 4 h after treatment, but the DNA repair capacity 24 h after treatment remained unaffected. These results suggest that MMR may stimulate induction (or conversion) of DNA lesions by heat, but is not involved in repair.

The excision repair pathways interplay at restoring DNA lesions induced by many different classes of chemotherapeutics, including alkylating agents and antimetabolites [104–107]. Hyperthermia sensitizes cells to many of these agents (Table 1), providing support for the hypothesis that excision repair pathways are affected by heat. However, clear interpretation of experimental and clinical data is hampered by extensive overlap of these mechanisms during repair of various lesions. For instance, DNA damage caused by alkylating agents, either directly or during processing of the initial lesions, can be repaired by NER, BER, MMR, as well as by SSB and DSB repair pathways [92, 105, 108] (Table 1).

**Table 1:** DNA damaging chemotherapeutic agents interacting with hyperthermia

Class	Agent [with references to studies showing interaction of the agent with hyperthermia]	Type of inflicted DNA damage	Pathways involved in repair [references]
Alkylating agents	- triazenes (temozolomide [182, 183])	strand cross-links, adducts, DSBs (indirect)	NER, BER, MMR, NHEJ, HR [108, 184]
	- nitrogen mustard derivatives (cyclophosphamide [13, 185–191], melphalan [191–199])		
	- aziridine-containing (mitomycin C [10, 187, 191, 200–203])		
Alkylating-like platinum compounds	- cisplatin [12, 100, 101, 191, 201, 204–210], carboplatin [211–214], oxaliplatin [198, 199, 209, 215]	strand cross-links, DSBs (indirect)	NER, BER, MMR, HR [94, 95, 216, 217]
Antimetabolites	- pyrimidine analogs (5-fluorouracil [218], gemcitabine [161, 199, 219])	SSBs, DSBs (indirect), oxidative damage	HR, MMR, NER [148, 161, 220, 221]
	- purine analogs (2-aminopurine [222], 6-thioguanine [222])		
	- dihydrofolate reductase inhibitors (methotrexate [210, 223])		
Topoisomerase I poisons	- camptothecin [224], B-lapachone [144, 145], Irinotecan [199]	SSBs	BER, NER, NHEJ [225, 226]
Topoisomerase II poisons	- intercalators (doxorubicin [187, 188, 227–230])	DSBs	NHEJ, HR [231–233]
Radiomimetics	- enediynes (neocarzinostatin [10])	SSBs, DSBs, oxidative damage, strand cross-links	HR, NHEJ, BER, [136, 137, 234, 235]
	- bleomycin [6, 10, 12, 191, 210, 236, 237]		
	- mitomycin C [10, 187, 191, 200–203]		
PARP inhibitors	- olaparib [150, 153], PJ-34 [150]	SSBs, DSBs (indirect)	HR, BER [238–240]

### Non-homologous end joining

Non-homologous end joining (NHEJ) is one of the major pathways to repair DSBs in mammalian cells. NHEJ is active throughout the cell cycle and rejoins the broken DNA ends without the requirement for homology or repair template [109]. Recently, two NHEJ subpathways have been discerned: the classical and alternative (or backup) NHEJ (alt-NHEJ). During the classical NHEJ (c-NHEJ), the Ku heterodimer is among the first factors that bind DNA ends. Upon binding, it becomes a scaffold for the subsequent recruitment of the end processing nucleases and ligases. As naturally occurring DSBs rarely result in clean DNA ends suitable for direct ligation, they are first processed by the Artemis/DNA-PKcs complex that provides various nucleolytic activities, and possibly by APLF and PNK. Ligation is then performed by the XLF/XRCC4/DNA ligase IV complex and the recently discovered XLF/XRCC4 paralog PAXX [110]. The Ku and ligase IV-independent alternative NHEJ may instead involve PARP1, XRCC1 and DNA ligases I or III [111]. While c-NHEJ is generally an accurate pathway, alt-NHEJ may be responsible for improper repair and formation of chromosome translocations in the absence of c-NHEJ [112, 113].

Whether NHEJ is inhibited by hyperthermia has been a subject of long debate. The initial evidence of NHEJ involvement can be found in data showing that hyperthermia sensitizes cells to ionizing radiation in G_1_-and G_0_-phase of the cell cycle, where mostly NHEJ mechanisms are responsible for repair of DSBs, although the degree of sensitization is increased in S- and M-phases [53, 114–116]. Later studies compared the degree of hyperthermia-mediated radiosensitization in wild-type and repair deficient Xrs-5 cells in plateau phase of growth and found that these cells could no longer be radiosensitized by hyperthermia. The rationale behind these experiments was that if repair pathway X is an (exclusive) target of hyperthermia, (wild-type) cells with a proficient pathway X can be radiosensitized by hyperthermia. This is in contrast to cells with a defect in pathway X which would no longer be radiosensitized. Using this logic, it was concluded that the DNA repair pathway defective in Xrs-5 cells is targeted by 43–45 °C hyperthermia [117, 118]. The deficiency in Xrs-5 cells was later attributed to the absence of a functional Ku protein [119–121], indirectly implicating NHEJ in heat-mediated radiosensitization. However, a number of subsequent studies showed no significant difference in sensitization of wild-type and NHEJ-deficient cell lines at similar temperatures (42.5-45.5 °C) [122–126]. Further, chemical inhibition of DNA-PK activity potentiated hyperthermia-mediated radiosensitization [127] and stimulated heat–induced apoptosis [128]. To explain this discrepancy, it was proposed that the Ku-independent alt-NHEJ pathway may instead be targeted by heat [129]. In log-phase cells, both c-NHEJ and alt-NHEJ pathways are active. Thus, in log-phase c-NHEJ-deficient cells, alt-NHEJ is still operational and, if this pathway is heat-sensitive, such cells could potentially be further sensitized by hyperthermia. In contrast, in plateau-phase cells alt-NHEJ seems severely compromised [130] and innate c-NHEJ deficiency would render such cells resistant to further heat-induced radiosensitization.

Although evidence of direct effects of hyperthermia on alt-NHEJ is lacking, effects on c-NHEJ factors have been observed by several groups. Studies showed heat-mediated inactivation of DNA binding by Ku and decreased activity of DNA-PK complex that correlated with the degree of radiosensitization, at temperatures of 44–45 °C [131–133]. Additionally, incubation at 44.5 °C induced aggregation of Ku in nuclei of human cells [134]. A recent study confirmed reversible repression of DNA-PK activity by 44 °C hyperthermia and reported considerable decrease in KU70 and KU80 protein levels, along with a more modest decrease in levels of BRCA1 and 53BP1 [135].

Hyperthermia (>41.5 °C) sensitizes cells to radiomimetic drugs such as bleomycin and neocarzinostatin that induce DSBs repaired by NHEJ mechanisms [6, 12, 136–139] (Table 1). One caveat in the interpretation of these experiments is that such drugs also induce SSBs and oxidative damage that may be repaired by other mechanisms or converted to DSBs and repaired by homologous recombination (see next paragraph) in the ensuing S/G_2_-phase of the cell cycle. Interestingly, even though DSBs indirectly induced by inhibitors of Topoisomerase II are primarily repaired by NHEJ [140], heat not only fails to sensitize cells to Topoisomerase II inhibitor etoposide, but exerts protective effects [141]. It has been suggested that hyperthermia may prevent formation of Topoisomerase II cleavage complexes after etoposide treatment, thereby reducing the DSB burden in treated cells [141]. This is in contrast to Topoisomerase I and II inhibitor β-lapachone [142, 143], whose cytotoxicity is potentiated by 42 °C hyperthermia [144, 145].

Thus, although indirect genetic studies do not confirm NHEJ as an exclusive target of hyperthermia, other results clearly support the notion that NHEJ is among the affected DNA repair mechanisms.

### Homologous recombination

Homologous recombination (HR) is the second DSB repair pathway of major importance in mammalian cells. HR requires a homology template, usually the sister chromatid, and is thus only active during S- and G_2_-phases. The first step in HR is the generation of 3’ single-stranded DNA overhangs, driven by the MRN complex. RPA quickly coats the exposed single-stranded DNA but is later replaced by RAD51 with help of BRCA2. The RAD51 nucleoprotein filaments are crucial for the search for the homologous duplex DNA, strand invasion, and the formation of the so-called Holliday junctions. The invading strand is extended by DNA polymerases, which copy the missing DNA sequence from the homologous template DNA, and, after dissolution of the Holliday junctions, the ends are ligated together [146].

Similarly to NHEJ, the involvement of HR in hyperthermic radiosensitization has been debated. Early studies indirectly excluded HR as a sole target of 42–45 °C hyperthermia [147], since rodent cell lines defective in XRCC2 and XRCC3, important HR factors, were normally radiosensitized by hyperthermia (43 °C) [80, 148], as were HR-deficient chicken DT40 cells (44 °C) [147, 149]. However, more direct readouts of HR later showed that hyperthermia (>41 °C) does inhibit HR, in human and mouse cells [150]. In particular, heat delays formation of IRIF by key HR proteins RAD51 and BRCA2 and inhibits HR-mediated gene targeting in mouse ES cells, possibly by inducing robust but temporary degradation of BRCA2 [150–152]. This hyperthermia-induced HR deficiency is enhanced by concomitant inhibition of HSP90 and can be used to sensitize cells to inhibitors of Poly (ADP-ribose) polymerase (PARP) [150, 153]. Heat (>41 °C) also inactivates RPA [154], reduces the levels of nuclear MRE11 protein and disrupts the interactions between the members of the MRN complex [155–158], which may be of consequence for initiation and progression of HR [159]. Interestingly, a reduction of BRCA1 protein levels is also seen upon heat exposure (42–44 °C) [135, 160] and BRCA1 seems to protect cells from effects of heat, such that overexpression of wild-type BRCA1 in cells decreases their heat sensitivity and mutant BRCA1 cells are more sensitive to treatment at 42 °C [160]. Additionally, the temperature of 42.5 °C may inhibit the recruitment of RAD51 to stalled replication forks [161].

Further evidence of targeting HR by hyperthermia can be found in studies of hyperthermic sensitization to various chemotherapeutic drugs. Nucleoside analogue gemcitabine is incorporated into the DNA during replication, leading to collapse of replication forks and generation of DSBs that are mostly restored by HR [148, 161, 162]. Hyperthermia (42.5 °C) inhibits the recruitment of RAD51 and impairs HR repair at stalled replication forks, thereby sensitizing cells to gemcitabine [161] (Table 1). HR is also involved in repair of SSBs and DSBs induced by ionizing radiation and other types of DNA damage, including cross-links induced by platinum compounds or mitomycin C, and hyperthermia can sensitize cells to all these agents (Table 1). However, multiple other pathways participate in repair of these lesions (Table 1), obscuring the importance of HR in the process.

### Clinical perspective

The potential of hyperthermia to sensitize (cancer) cells to DNA damaging agents (Table 1) has been obvious for many decades. However, clear clinical benefits could only be demonstrated much later, perhaps due to technical challenges related to the development of reliable hyperthermia applicators, treatment planning and adequate dosimetry [163–165]. The effectivity of hyperthermia combined with radiation has been demonstrated in several randomized phase II/III trials for melanoma, cervix, breast, head and neck cancer, showing a significant enhancement in radiation effectivity without a significant increase in toxicity [166–170]. Also, the combination of hyperthermia and cisplatin or similar agents has been tested in a number of phase II and some phase III trials. Hyperthermia enhanced the effectiveness of mitomycin C in phase III trials for bladder cancer [171, 172] and of etoposide, ifosfamide and doxorubicin for soft tissue sarcomas [173]. A review on Hyperthermic IntraPEritoneal Chemotherapy (HIPEC) treatment for ovarian cancer showed no increase of toxicity due to hyperthermia [174]. Reviews summarizing about 30 randomized hyperthermia trials are given in [175–177]. An overview of the clinical effectivity and toxicity of trimodality treatment schedules comprising hyperthermia, radiation and cisplatin or oxaliplatin was given by [178], listing 13 nonrandomized phase I/II trials for breast, head and neck, cervix and oesophagus cancer. Results showed that this form of trimodality treatment is feasible and effective with only moderate toxicity. Also, multiple studies in recurrent cervical cancer show that hyperthermia enhanced the uptake and cytotoxicity of cisplatin without additional side effects [19, 179–181]. Summarizing, hyperthermia has shown very significant enhancement of the effectivity of both radiotherapy and chemotherapy without increasing toxicity in various multi-modality settings. The multitude of drug combinations and treatment modalities that show positive effects in combination with hyperthermia seems to reflect the multitude of DNA repair and other pathways that are affected by heat.

## Conclusions

**Fig. 1:**
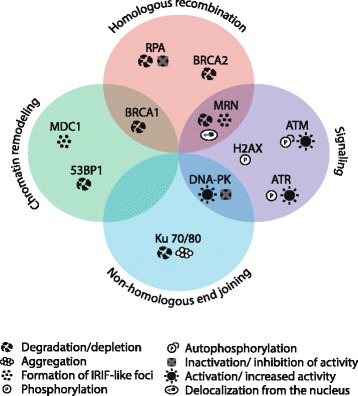
Schematic overview of the effects of hyperthermia on DNA repair factors BRCA1 [135, 160], BRCA2 [150], MRN complex [30, 37, 155–158], RPA [71, 154], ATM [35, 36, 43], ATR [67, 68], DNA-PK [43, 133, 135], Ku70/80 [131, 132, 134, 135], H2AX [31, 37, 38], MDC1 [35] and 53BP1 [135]

Hyperthermia has been subject of investigations for nearly half a century, yet its numerous effects on cells and tissues still remain unclear. In particular, it is not well known how heat interacts with DNA repair pathways, which is highly relevant in clinical cancer treatment. It is apparent from studies reviewed here that in the early years of hyperthermia research many of major effects of hyperthermia on cells were observed, but mechanistic insight was lacking due to limited understanding of cellular pathways, including DDR. As this understanding deepened and new molecular biology tools became available in the 1990s and 2000s, the search for proteins and pathways targeted by hyperthermia intensified. Major contributions were made by studies that analysed hyperthermic sensitization in DNA repair-deficient cells. However, results of these studies were generally interpreted under assumption that one major pathway is responsible for the effects of heat on DNA repair, leading to multiple conflicting hypotheses. We now only begin to see how many facets of DDR are disturbed, including direct effects on major DNA repair factors (Fig. 1), damage signaling, checkpoints, cell cycle progression and apoptosis.

Although difficult to study, these effects are highly beneficial in clinical practice. By disturbing multiple DNA repair pathways, hyperthermia sensitizes cells to a broad range of DNA-damaging agents. Recent clinical trials clearly demonstrated the benefits and safety of treatments involving hyperthermia. Although much remains to be discovered, hyperthermia is no longer the black box it once was and it is bound, in the near future, to take more central stage in clinical cancer treatment.
